# Factors associated with the export of traditional Chinese medicinal products: A stochastic frontier analysis

**DOI:** 10.1371/journal.pone.0326422

**Published:** 2025-07-09

**Authors:** Ying Chen, Shuduo Zhou, Zhongfei Pei, Weili Zhao, Jian Yang, Yunxuan Hu, Xiangning Feng, Ming Xu

**Affiliations:** 1 Department of Global Health, School of Public Health, Peking University, Haidian District, 38 Xue Yuan Road, Beijing, China; 2 Chinese Medical Association, Dongcheng District, 42 Dong Si Xi Road, Beijing, China; University of Minnesota - Duluth, UNITED STATES OF AMERICA

## Abstract

**Background:**

The export of traditional Chinese medicinal products is a pivotal force in enhancing the international recognition of Traditional Chinese Medicine (TCM). From 2013 to 2022, the export trend of traditional Chinese medicinal products demonstrated stable and rapid growth. However, the export structure remained suboptimal, with raw material-based products accounting for a significantly larger proportion than high value-added products. This imbalance has hindered the enhancement of traditional Chinese medicinal value and the potential for branding in the global market. Therefore, an in-depth study of the current status and associated factors of the export is essential to optimize the export structure, adjust export policies, and promote the international trade.

**Methods:**

We employed joinpoint regression analysis to examine the export value trend of traditional Chinese medicinal products from 2013 to 2022. Subsequently, we utilized stochastic frontier analysis and trade inefficiency analysis to investigate the factors associated with the export value across 182 countries and regions from 2020 to 2022. The export value data was sourced from the China Chamber of Commerce for Import and Export of Medicines and Health Products. The associated factors were sourced from publicly available databases.

**Results:**

Joinpoint regression analysis showed the Annual Average Percent Change (AAPC) of the export value of traditional Chinese medicinal products from 2013 to 2022 was 6.9211 (95% Confidence Interval [CI] 5.9176 to 7.9341). Among them, raw material-based products included Chinese medicinal materials and herbal decoction pieces, as well as plant extracts, with AAPC being 1.7822 (95%CI −3.4203 to 7.2649), and 10.0290 (95%CI 3.5599 to 16.9023), respectively. They comprised over 80% of total exports. High value-added products included Chinese patent medicines and TCM health products, with AAPC being 3.6420 (95%CI −0.7595 to 8.2387) and 17.0895 (95%CI 4.7468 to 30.8865), respectively. They constituted a relatively minor share of total exports. Stochastic frontier analysis showed the coefficient (t-ratio) of factors associated with the export, including China’s GDP at 0.0772 (9.3276), the GDP of the export country or region at 0.1240 (3.9521), the capital distance between the export country or region and China at −0.3059 (−8.2793), the population of the export country or region at 0.0329 (3.4657), having a common border with China at −0.3179 (−4.3428), having a common language with China at 1.3316 (13.3217), being a landlocked country or region at −0.3531 (−9.3519). Trade inefficiency analysis showed the coefficient (t-ratio) of associated factors, including having a trade agreement with China at −7.1587 (−38.7960), pharmaceutical products tariffs at 0.2930 (7.7920), cultural distance from China at 0.5995 (7.1274), the overseas registration status of Chinese patent medicines at 2.3608 (13.6945), and the presence of TCM institutions at 6.1643 (16.1009).

**Conclusions:**

The export value of traditional Chinese medicinal products from 2013 to 2022 showed an overall upward trend. However, the export structure remained suboptimal. Stochastic frontier analysis and trade inefficiency analysis revealed the relevance of associated factors to the export value. Based on the above results, we recommend six measures to optimize the export structure and enhance the international recognition of traditional Chinese medicinal products.

## Introduction

Traditional Chinese Medicine (TCM) is integral to China’s cultural heritage and has been disseminated to over 180 countries and regions worldwide [1]. The export of traditional Chinese medicinal products plays a crucial role in the international development of TCM. From 2013 to 2022, the export value of these products experienced steady and rapid growth, increasing from USD 3.14 billion in 2013 to USD 5.69 billion in 2022, with a compound annual growth (CAGR) rate of 6.8% [2]. Despite this growth, the export structure remained suboptimal, with raw material-based products dominating the market and accounting for over 80% of the total export value. In contrast, high value-added products, such as Chinese patent medicines, comprised only about 8% of exports. The export of Chinese patent medicines grew slowly, showing a trade deficit and limiting traditional Chinese medicinal products’ value enhancement and branding in the international market. China is endowed with abundant traditional Chinese medicinal resources. However, these have not been fully leveraged to create competitive advantages in the global market [3]. The differences in theoretical foundations between TCM and western medicine pose significant challenges to the international acceptance of TCM. Currently, most traditional Chinese medicinal products are not included in European countries’ health insurance systems, and most Chinese patent medicines are marketed overseas as food or dietary supplements.

Several researchers examined the factors associated with the export values of traditional Chinese medicinal products. They usually analyzed data from several countries or regions, with associated factors including China’s gross domestic product (GDP), the GDP of the export country, China’s population, the population of the export country, capital distance, the presence of regional trade agreements, free trade agreements, economic disparities between countries, cultural distance, and the presence of Confucius Institutes, etc [4–7]. Various methodologies, such as the gravity model, extended gravity model, and stochastic frontier analysis were employed to analyze the associated factors. While these selected countries and regions were representative of previous studies, they did not provide a comprehensive overview of the global export situation. Descriptive analysis was mainly used to assess the current status, with a notable lack of trend analysis of the overall data. The variables included in these analyses were generic factors associated with export trade development. However, there was a lack of specific variables pertinent to the export of traditional Chinese medicinal products, such as the registration status of Chinese patent medicines and the establishment of overseas TCM institutions. Although one study included the association of Confucius Institutes on the export [7], there was a significant gap in the analysis of other TCM institutions. These include overseas TCM centers, overseas branches of TCM universities, and TCM hospitals. A comprehensive understanding of these associated factors is essential for developing targeted strategies to enhance the international recognition of TCM.

This study employed joinpoint regression to analyze the trend in the export value of traditional Chinese medicinal products from 2013 to 2022. Subsequently, we utilized stochastic frontier analysis (SFA) and trade inefficiency analysis to examine the factors associated with the export value of traditional Chinese medicinal products across 182 countries and regions from 2020 to 2022. An in-depth analysis of the current status and associated factors of traditional Chinese medicinal products export is crucial. Such a study can provide suggestions to optimize the export structure, adjust export policies, and facilitate the integration of traditional Chinese medicinal products into the international pharmaceutical market. Ultimately, these efforts will enhance traditional Chinese medicinal’s global development and recognition.

## Methods

### 2.1 Joinpoint regression model principle

The joinpoint regression model, initially proposed by Kim et al. in 1998 [8], is designed to segment long-term trends into distinct intervals, each described by a continuous log-linear model. This segmentation enables the identification of various phases within the trend, including upward, rapid upward, stable, downward, and rapid downward segments. Such differentiation allows researchers to investigate the underlying causes and contexts of these trends. It has been extensively utilized for analyzing trends in tumor incidence, mortality, and survival rates.

The joinpoint regression model is a log-linear model based on the Poisson Distribution. It utilizes rate values, such as morbidity, mortality, and survival rates, as dependent variables, with time (in years) serving as the independent variable. The model employs the Monte Carlo Permutation Test for hypothesis testing to detect significant changes in the overall trend of rate values. It identifies turning points, segmental change points, or connectors to delineate the long-term trend of these rates. The model quantifies the magnitude of the trend within each time segment using the Annual Percent Change (APC). The equation for the joinpoint regression model is [9]:


$$y_{i}=\beta_{0}+\beta_{1}\cdot t_{i}+\sum_{k=1}^{k}\delta_{k}\cdot s_{k}(t_{i})+\varepsilon_{i}$$ (1)

$$y=\beta_{0}+\beta_{1}\cdot t+\delta_{1}\cdot \left(t-\tau_{1}\right)\ldots +\delta_{k}\cdot \left(t-\tau_{k}\right)$$ (2)

where, $y_{i}\left(i=1,\;2,\;3,\;\ldots ,\;n\right)$ is the dependent variable, representing the log rate values of morbidity, mortality, or survival. $t_{i}\left(i=1,\;2,\;3,\;\ldots ,\;n\right)$ is the independent variable, representing the time of the incident corresponding to this set of log rate values. $s_{k}(t_{i})={\left(t-\tau_{k}\right)}^{+}$ is the segmented function, with ${\left(t-\tau_{k}\right)}^{+}=t-\tau_{k}$ when $\left(t-\tau_{k}\right)>0$, and ${\left(t-\tau_{k}\right)}^{+}=0$ when $\left(t-\tau_{k}\right)\leq 0$. $\tau_{i}\left(1,\;2,\;3,\;\ldots ,\;k\right)$ represents the linkage points, and *k* is the number of linkage points. $\left(\beta_{0},\;\beta_{1},\;\delta_{1},\;\ldots \;,\;\delta_{k0},\;\tau_{1},\;\ldots ,\;\tau_{k0}\right)$ are the parameters of the regression model. $\varepsilon_{i}$ is the random error.

Recently, its application has expanded to analyze drug usage, product sales, and shifts in research trends within specific fields [10–12]. In this study, Joinpoint Regression Program Version 5.0 was utilized to analyze the patterns of change in the export value of traditional Chinese medicinal products from 2013 to 2022, and to identify turning points in the export value trend. The Annual Percent Change (APC) and Annual Average Percent Change (AAPC), along with their 95% Confidence Intervals (CIs), were calculated. An APC or AAPC > 0 indicates an increasing trend in the data during the specified period, while an APC or AAPC < 0 signifies a decreasing trend. When APC = AAPC, it indicates a monotonic increase or decrease in the trend [9]. The test level is alpha = 0.05, with 95% CI, not including 0, being considered statistically significant. If the result is not statistically significant, it suggests that the change trend is relatively stable [13]. All relevant data were sourced from the China Chamber of Commerce for Import and Export of Medicines and Health Products (CCCMHPIE). CCCMHPIE classifies traditional Chinese medicinal products into four subcategories: Chinese medicinal materials and decoction pieces, Chinese patent medicine, plant extracts, and TCM health products.

### 2.2 Stochastic frontier analysis model principle

The traditional gravity model, first applied to economics by Tinbergen (1962) and Poyhonen (1963), demonstrated that total trade was associated with geographic location and economic aggregation. Subsequent Linnmann (1966) and Bergstrand (1989) expanded the model by incorporating additional factors such as population and trade agreements. The standard gravity model was finally derived by Evenett and Keller (2002). However, many traditional gravity models employs ordinary least squares (OLS) to estimate trade efficiency and potential, which only reflects average effects of associated factors and is prone to significant estimation errors.

To address these limitations, Aigner (1977) and Meeusen (1977) introduced stochastic frontier analysis into the gravity model framework. Unlike traditional gravity models, the SFA model uses maximum likelihood estimation (MLE) to calculate trade efficiency and potential, resulting in more precise estimates with reduced error. The SFA model also incorporates a range of realistic and variable trade associated factors that can impede trade development, enhancing its accuracy in estimating trade efficiency and potential [6].

Battese and Coelli (1992, 1995) further advanced the SFA model with the development of the BC92 and BC95 models. The BC92 model, also known as the stochastic frontier analysis model with time-varying inefficiency, is designed for use with unbalanced panel data. It assumes that trade inefficiency follows a truncated normal distribution and allows for variation over time. Its basic form is as follows:


$$Y_{it}=X_{it}\beta +\left(\nu_{it}-\mu_{it}\right),i=1,\;2,\;3,\;\ldots ,\;N;t=1,\;2,\;3,\;\ldots ,\;T$$ (3)

$$\mu_{it}=\mu_{i}\exp\left[-\eta \left(t-T\right)\right]$$ (4)

in equation (3), $Y_{it}$ represents the actual trade volume in the period t. $X_{it}$ represents the natural factors affecting trade. *β* represents the parameter estimated in the regression model; *v* represents the random error term in the model; $\mu_{it}$ represents the trade inefficiency term.

In equation (4), exp[−η(t−T)]≥0,$\mu_{it}$ obeys a half-normal distribution and η is a time-varying parameter to be estimated. When η=0, it means that $\mu_{it}$ does not vary with time, and the model is a stochastic frontier analysis model that does not vary with time. When η>0, it means that $\mu_{it}$ decreases with time. When η<0, it means that $\mu_{it}$ increases with time.

The BC92 model estimated trade efficiency in a two-stage process, which could result in discrepancies between the outcomes of the two stages. To address this, Battese and Coelli (1995) proposed the BC95 model, which allowed for single-stage estimation of trade efficiency and variable coefficients. This model directly regresses the associated factors of trade inefficiency [6]. The trade inefficiency term in the BC95 model is defined as follows:


$$\mu_{it}=\alpha Z_{it}+\delta_{it}$$ (5)

in equation (5), $Z_{it}$ refers to the associated factors of trade inefficiency. α is the model parameter. $\delta_{it}$ is the random perturbation term. When α>0, it means that the variable can promote trade inefficiency and it hinders the development of trade. When α<0, it means that the variable can suppress trade inefficiency and it promotes the development of trade.

SFA is a method used to measure production efficiency and cost efficiency. Other models or methods with similar functions to SFA mainly include Data Envelopment Analysis (DEA) and Total Factor Productivity (TFP) analysis. The unique advantage of SFA lies in its ability to distinguish between efficiency loss and random error while offering stronger statistical testing capabilities. This makes SFA particularly useful for efficiency analysis in industries such as agriculture, banking, and healthcare, especially when random external factors have significant impacts. Additionally, the model flexibility of SFA and its applicability to small samples provide further advantages for its use in specific contexts.

Frontier 4.1 software was employed in this study to analyze the export data of traditional Chinese medicinal products from 182 countries and regions. Due to changes in the Custom Harmonized System (HS) Codes, this study focused on the export data of traditional Chinese medicinal products from 2020 to 2022. Referring to previous studies [14–16], this study analyzed the factors associated with the export value of traditional Chinese medicinal products, including China’s GDP, China’s population, the GDP of the export country or region, the population of the export country or region, the presence of a common language with China, the capital distance between the export country or region and China, the presence of a common border with China, the landlocked status of the export country or region, the existence of a trade agreement with China, pharmaceutical products tariffs in the export country or region, cultural distance from China, the overseas registration status of Chinese patent medicines, and the presence of TCM institutions in the export country or region. Data on the export value of traditional Chinese medicinal products were sourced from CCCMHPIE. The sources of the associated factors and the expected results were detailed in S1 Table. The calculation of cultural distance followed the methodology outlined in 0 study [17], using the most recent data on the six cultural dimensions provided by the Hofstede official website:


cul=∑u=1u(Iju−Iiu)2/(Iju−Iiu)2Vu\nulldelimiterspaceVuu (6)

in equation (6), *i* represents China, *cul* represents the cultural distance between China and *j* country or region, $I_{ju}$ represents the score of *j* country or region on the *u* cultural dimension, $I_{iu}$ represents the score of China on the *u* cultural dimension, and $V_{u}$ represents the variance of *u* cultural dimension.

The joinpoint regression analysis identified significant trend shifts in the export value of traditional Chinese medicinal products, which informed the SFA by providing a basis for examining how these structural changes, along with other economic and policy factors.

## Results

### 1. Joinpoint regression analysis results

**Fig 1:**
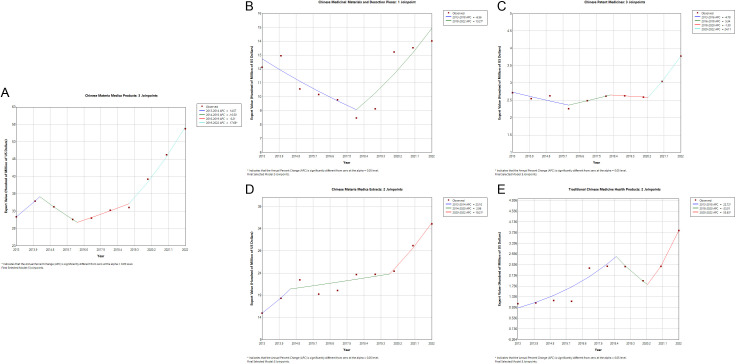
Joinpoint regression of the export value of traditional Chinese medicinal products (2013-2022) in subcategories.

The total export value of traditional Chinese medicinal products showed a significant upward trend from 2013 to 2022, with AAPC being 6.9211 (95%CI 5.9176 to 7.9341), which 95%CI was statistically significant. It was categorized into four periods. From 2013 to 2014, APC was 14.5700 (95%CI −0.5535 to 31.9933). From 2014 to 2016, APC was −10.4998 (95%CI −22.6930 to 3.6165). From 2016 to 2019, APC was 6.2082 (95%CI −8.3472 to 23.0752). The 95%CIs for the growth rates above were not statistically significant, suggesting a relatively stable overall change in the export value. However, a significant upward trend in the export value of traditional Chinese medicinal products was observed from 2019 to 2022, with APC being 17.6838 (95% CI 12.0936 to 23.5528), which 95%CI was statistically significant, as shown in Fig 1a.

For Chinese medicinal materials and decoction pieces, the total export value from 2013 to 2022 was divided into two periods. 95%CI of AAPC was not statistically significant. From 2013 to 2018, APC was −6.5616 (95%CI −14.1473, 1.6943), which 95%CI was not statistically significant, suggesting a relatively stable overall change in the export value. From 2018 to 2022, APC was 13.2673 (95%CI 1.1644 to 26.8182), which 95%CI was statistically significant, indicating that the export value showed a significant upward trend, as shown in Fig 1b.

For Chinese patent medicines, the total export value from 2013 to 2022 was divided into four distinct periods. From 2013 to 2016, APC was −4.7756 (95%CI −32.1480, 33.6392). From 2016 to 2018, APC was 5.3367 (95%CI −48.2203, 114.2888). From 2018 to 2020, APC was −1.3011 (95%CI −50.7067, 97.6225). From 2020 to 2022, APC was 24.1078 (95%CI −27.7432, 113.1668). The 95%CIs for these periods were not statistically significant. The overall AAPC was 3.6420 (95% CI −0.7595 to 8.2387), which 95%CI was not statistically significant. This indicated a relatively stable change over the period, as shown in Fig 1c.

For plant extracts, the total export value from 2013 to 2022 exhibited a significant upward trend, with AAPC being 10.0290 (95%CI 3.5599 to 16.9023), which 95%CI was statistically significant. It was divided into three periods. From 2013 to 2014, APC was 23.1039 (95%CI −19.1646, 87.4742). From 2014 to 2020, APC was 2.9564 (95%CI −3.8031, 10.1909). The 95% CIs for these periods were not statistically significant. From 2020 to 2022, APC was 19.2130 (95%CI 5.8439, 34.2707), which 95%CI was statistically significant, it demonstrated a significant upward trend, as shown in Fig 1d.

For TCM health products, the overall export value from 2013 to 2022 demonstrated a significant upward trend, with AAPC being 17.0895 (95%CI 4.7468 to 30.8865), which 95%CI was statistically significant. It was divided into three periods. From 2013 to 2018, APC was 22.7203 (95%CI 7.3414 to 40.3026). From 2020 to 2022, APC was 53.6300 (95%CI 1.8900 to 131.6439). Both periods showed statistically significant increases, indicating a marked upward trend. While, from 2018 to 2020, APC was −23.0072 (−55.9916, 34.6990), which 95%CI was not statistically significant, as shown in Fig 1e. Detailed information was provided in Table 1.

**Table 1:** APC and AAPC of joinpoint regression of the export value of traditional Chinese medicinal products (2013-2022) in subcategories.

Category	Export Value (a hundred million in US Dollars)		
**Period (Year)**	**APC (95%CI)**	**AAPC (95%CI)**
Traditional Chinese Medicinal Products (Total)	2013-2014	14.5700 (−0.5535, 31.9933)	6.9211^△^ (5.9176, 7.9341)
	2014-2016	−10.4998 (−22.6930, 3.6165)	
	2016-2019	6.2082 (−8.3472, 23.0752)	
	2019-2022	17.6838^*^ (12.0936, 23.5528)	
Chinese Medicinal Materials and Decoction Pieces	2013-2018	−6.5616 (−14.1473, 1.6943)	1.7822 (−3.4203, 7.2649)
	2018-2022	13.2673^*^ (1.1644, 26.8182)	
Chinese Patent Medicines	2013-2016	−4.7756 (−32.1480, 33.6392)	3.6420 (−0.7595, 8.2387)
	2016-2018	5.3367 (−48.2203, 114.2888)	
	2018-2020	−1.3011 (−50.7067, 97.6225)	
	2020-2022	24.1078 (−27.7432, 113.1668)	
Plant Extracts	2013-2014	23.1039 (−19.1646, 87.4742)	10.0290^△^ (3.5599, 16.9023)
	2014-2020	2.9564 (−3.8031, 10.1909)	
	2020-2022	19.2130^*^ (5.8439, 34.2707)	
TCM Health Products	2013-2018	22.7203^*^ (7.3414, 40.3026)	17.0895^△^ (4.7468, 30.8865)
	2018-2020	−23.0072 (−55.9916, 34.6990)	
	2020-2022	53.6300^*^ (1.8900, 131.6439)	

* Indicates that the APC is significantly different from zero at the alpha = 0.05 level.

△Indicates that the AAPC is significantly different from zero at the alpha = 0.05 level

## 2. Stochastic frontier analysis and trade inefficiency analysis results

### 2.1 Missing value response

Countries or regions with excessive missing values were excluded from the analysis. For those with a limited number of missing values, the panel data were interpolated using multiple imputation methods. Multiple imputation is a statistical technique for handling missing values in datasets, proposed by Donald Rubin in 1978. This method not only accounts for the uncertainty of individual missing values, but also reflects this uncertainty by creating multiple complete datasets with different imputed values. Each dataset is generated by estimating and simulating the missing values in the original data, allowing for independent analysis of each dataset. The final statistical inference is obtained by aggregating the results from these analyses, incorporating the uncertainty introduced by the missing values. In our study, multiple imputation resulted in five distinct interpolated datasets. The reliability of these interpolated datasets was then assessed. The dataset with the highest reliability was selected for further analysis.

### 2.2 Multicollinearity test

To mitigate the risk of pseudo-regression due to multicollinearity arising from high correlations between variables, the Variance Inflation Factor (VIF) was employed to assess multicollinearity. The results revealed that the VIF for both China’s GDP and China’s population exceeded 5, indicating the presence of multicollinearity between these variables. Following the exclusion of the population variable, the VIF values for all remaining variables were below 5, thereby eliminating multicollinearity.

### 2.3 Serial correlation test

Due to the small time dimension of the data, the Wooldridge test was used for the serial correlation test. The results showed that, H0: no first-order autocorrelation, F(1,181)=1.282, P = 0.2589 > 0.05. These results indicated that the null hypothesis could not be rejected, and there was no significant serial correlation.

### 2.4 Stochastic frontier analysis applicability test

The Likelihood Ratio (LR) test was employed to evaluate the model’s applicability [18], specifically addressing the existence of a trade inefficiency term, the potential time variation, and the inclusion of specific variables. The variables tested included the GDP of the export country or region (*gdp*), China’s GDP (*cgdp*), the population of the export country or region (*pop*), capital distance (*dis*), common border status (*bor*), common language (*lan*), and landlocked status (*loc*). The results of the hypothesis testing were as follows: (1) The hypothesis was rejected that the trade inefficiency term was absent, confirming the suitability of using stochastic frontier analysis for this study. (2) The hypothesis was rejected that the trade inefficiency term was static over time, supporting a time-varying stochastic frontier analysis model. (3) The hypotheses excluding *gdp*, *cgdp*, *pop*, *dis*, *bor*, *lan*, and *loc* were rejected, indicating that these variables were appropriately included in the model, as detailed in S2 Table.

### 2.5 Stochastic frontier analysis results

This study employed SFA to examine the factors associated with the export value of traditional Chinese medicinal products across 182 countries and regions from 2020 to 2022. To assess the robustness of the estimation results, we compared the outcomes of both time-invariant and time-varying SFA models, as presented in Table 2. The parameter γ, representing the proportion of trade inefficiency in the stochastic error term, exceeded 0.9 in both models and was statistically significant at the 1% level. The signs and significance of the variables were generally consistent across both models, with exceptions noted for the constant term and China’s GDP in the time-invariant model. This consistency indicated the robustness of the SFA model, confirming that the trade inefficiency term existed and that the discrepancy between the actual trade value and the trade potential for traditional Chinese medicinal products was primarily due to trade inefficiency. Additionally, the time-varying coefficient η was significant at the 1% level, suggesting that the trade inefficiency term varied over time, demonstrating the greater applicability of the time-varying model compared to the time-invariant model. The negative value of η indicated a decline in trade efficiency for traditional Chinese medicinal products export over the study period.

**Table 2:** Stochastic frontier analysis results.

Variable	Time-Varying Model			Time-Invariant Models		
**Coefficient**	**Standard-Error**	**t-Ratio**	**Coefficient**	**Standard-Error**	**t-Ratio**
constant	−2.8091***	0.9881	−2.8428	−1.1216	0.8775	−1.2781
*lngdp*	0.0772***	0.0083	9.3276	0.0623***	0.0131	4.7635
*lncgdp*	0.1240***	0.0314	3.9521	0.0525*	0.0308	1.7027
*lndis*	−0.3059***	0.0369	−8.2793	−0.2317***	0.0346	−6.7004
*lnpop*	0.0329***	0.0095	3.4657	0.0600***	0.0158	3.8015
*lnbor*	−0.3179***	0.0732	−4.3428	−0.1854***	0.0611	−3.0360
*lnlan*	1.3316***	0.1000	13.3217	1.4128***	0.1854	7.6211
*lnloc*	−0.3531***	0.0378	−9.3519	−0.2849***	0.0949	−3.0010
σ2	0.1556***	0.0142	10.9864	0.0912***	0.0081	11.3129
γ	0.9843***	0.0014	721.5855	0.9791***	0.0017	572.9834
μ	0.3211***	0.0649	4.9452	0.5975***	0.0374	15.9888
η	−0.0251***	0.0072	−3.4997	–	–	–
log likelihood function	369.7711			415.7678		
LR test of the one-sided error	780.9166			872.9100		

***, **, * respectively represents 1%, 5% and 10% significance levels.

In the time-varying model, the coefficient for the *lngdp* variable was positive and statistically significant at the 1% level, indicating a substantial positive association between the export country’s or region’s GDP and the export of traditional Chinese medicinal products. Similarly, the *lncgdp* variable also exhibited a positive coefficient and was significant at the 1% level, demonstrating that China’s GDP was positively associated with the export. The *lndis* variable showed a negative coefficient and was significant at the 1% level, suggesting that the distance between the export country or region and China’s capital city was adversely associated with the export. The *lnpop* variable had a positive coefficient and passed the significance test at the 1% level, indicating that the population size of the export country or region was positively associated with the export. The coefficient for the *lnbor* variable was negative and statistically significant at the 1% level, revealing that having a common border with China was negatively associated with the export. Conversely, the *lnlan* variable had a positive coefficient and was significant at the 1% level, indicating that having a common language with China was positively associated with the export. The *lnloc* variable also exhibited a negative coefficient and was significant at the 1% level, demonstrating that being landlocked was negatively associated with the export. All variables, except for the *lnbor*, aligned with the expected results, confirming the overall consistency of the model’s findings.

### 2.6 Trade inefficiency analysis results

The SFA results confirmed the presence of a trade inefficiency term, which was shown to vary over time. Using the *one-stage* estimation method [6], we analyzed the factors associated with the trade inefficiency term, as detailed in Table 3. The parameter γ was greater than 0.9 and statistically significant, indicating that the SFA model was appropriately specified and that trade inefficiency was a significant impediment to the export of traditional Chinese medicinal products. The coefficient for the *lnwto* variable was negative and statistically significant at the 1% level, suggesting that trade agreements significantly reduced trade inefficiency, promoting the export of traditional Chinese medicinal products. Conversely, the *lncus* variable exhibited a positive coefficient and was significant at the 1% level, indicating that higher tariffs on pharmaceutical products increased trade inefficiency, thereby obstructing the export. The *lncul* variable also had a positive coefficient and was significant at the 1% level, showing that greater cultural distance impeded the export. These findings were consistent with the expected results. However, the *lnpat* and *lnins* variables had positive coefficients and were significant at the 1% level, suggesting that the overseas registration status of Chinese patent medicines and the presence of overseas TCM institutions were significantly positively associated with trade inefficiency. This indicated that these factors hindered rather than facilitated the export, contrary to the initial expectations.

**Table 3:** Trade inefficiency analysis results.

Model	Variable	Coefficient	Standard-Error	t-Ratio
SFA Model	constant	0.1180	0.0959	1.2298
	*lngdp*	0.0013^***^	0.0002	5.6421
	*lncgdp*	−0.0044	0.0031	−1.4053
	*lndis*	−0.0064^***^	0.0008	−7.5875
	*lnpop*	0.0026^***^	0.0002	12.7491
	*lnbor*	−0.0054^**^	0.0023	−2.3498
	*lnlan*	0.8670^***^	0.0009	1008.4786
	*lnloc*	−0.0037	0.0027	−1.3567
Trade Inefficiency Model	constant	−6.5276^***^	0.4786	−13.6378
	*lnwto*	−7.1587^***^	0.1845	−38.7960
	*lncus*	0.2930^***^	0.0376	7.7920
	*lncul*	0.5995^***^	0.0841	7.1274
	*lnpat*	2.3608^***^	0.1724	13.6945
	*lnins*	6.1643^***^	0.3829	16.1009
	σ2	0.6162^***^	0.0408	15.0978
	γ	0.9999^***^	0.00000009	11423545
	log likelihood function	637.3156		
	LR test of the one-sided error	1316.0055		

*** ,

** ,

* respectively represents 1%, 5% and 10% significance levels.

### 2.7 Robustness test of trade inefficiency analysis

We employed the variable exclusion method to assess the trade inefficiency analysis model’s robustness. Specifically, we sequentially removed the following variables: *lnwto*, *lncus*, *lncul*, *lnpap*, and *lnins*. The analysis demonstrated that the signs of the regression coefficients and the significance levels of the remaining variables generally remained consistent with the original results. However, notable exceptions were observed: the exclusion of the *lncus* variable rendered the *lnwto* variable statistically insignificant, and the exclusion of the *lnpap* variable resulted in the *lncul* variable losing significance and its sign reversing to negative. These findings indicated some sensitivity in the robustness of the results. For detailed results, refer to S3 Table.

## Discussion

### 1. Trade trend analysis

The results from the joinpoint regression analysis indicated a significant upward trajectory in the export value of traditional Chinese medicinal products from 2013 to 2022, with a notable inflection point in 2019. This turning point was closely associated with several policy advancements and shifts in the market environment. The *Belt and Road* proposal initiation in 2013 presented a new opportunity to export traditional Chinese medicinal products. During the initial phase (2013–2014), there was a steady increase in the export value. However, between 2014 and 2016, the export value experienced a decline, although the 95%CI was not statistically significant, suggesting a relatively stable variation. This period of decline might be attributed to fluctuations in the international market and changes in the global economic landscape. In 2016, the State Council’s release of the *Outline of the Strategic Plan for the Development of Traditional Chinese* Medicine (2016*–2030)* elevated the development of TCM to a national strategic priority. The same year also saw the publication of the *White Paper on Traditional Chinese Medicine in China* and the enactment of the *Law of the People’s Republic of China on Traditional Chinese Medicine*, which collectively enhanced the international prominence of TCM. These measures began to yield positive outcomes in subsequent years, resulting in relatively steady growth in the export value from 2016 to 2019. From 2019 onwards, particularly with the release of the *Development Plan for Promoting High-Quality Integration of Traditional Chinese Medicine into the Construction of the* Belt and Road (2021*–2025)* and the *14th Five-Year Plan for the Development of Traditional Chinese Medicine*, China’s TCM policy framework was further refined, providing substantial support for the export. This period (2019–2022) saw a significant rise in export value, reflecting the dual impact of favorable policy measures and increased market demand.

Despite the overall growth in the export value of traditional Chinese medicinal products, this increase was predominantly driven by raw material-based products. Chinese medicinal materials and decoction pieces, along with plant extracts, constituted over 80% of total exports. In contrast, Chinese patent medicines and TCM health products contributed a relatively minor share of the total export value. This composition indicated that the export market for traditional Chinese medicinal products remained in the primary raw materials stage, reflecting limited value addition and processing of Chinese medicines.

Examining export trends, the export value of Chinese patent medicines exhibited slow growth from 2013 to 2022, with 95% CIs indicating that these changes were not statistically significant, and overall stability characterized this period. Conversely, the export value of plant extracts increased significantly, surpassing that of Chinese medicinal materials and decoction pieces in recent years. This growth was primarily attributed to a global health trend, increased interest in green consumption, and rising market demand for essential oil products [5]. However, this growth was more reflective of shifting market demand rather than improvements in the overall competitiveness of the traditional Chinese medicinal industry. The differences in theoretical foundations between TCM and western medicine contribute to the limited international acceptance and recognition of traditional Chinese medicinal products. Traditional Chinese medicinal products have not attained legal status in many countries, particularly in developed countries, where the registration and use of Chinese patent medicines are tightly regulated. Consequently, these products are often marketed as food or dietary supplements rather than medicines, significantly constraining their market potential and value addition.

A comparison with global trade patterns in herbal medicine highlighted a key structural limitation of traditional Chinese medicinal products exports. In contrast to Japan and South Korea, which had successfully commercialized their traditional medicine industries by integrating standardized formulas and modern production techniques into pharmaceutical-grade products, traditional Chinese medicinal products exports were still primarily unprocessed or minimally processed [19]. Similarly, Germany and other European countries had established robust herbal medicine industries by emphasizing clinical validation, standardized extraction techniques, and stringent quality control [20]. These factors had enabled European phytopharmaceuticals to secure higher market recognition and value addition compared to traditional Chinese medicinal products, which were often categorized as dietary supplements in foreign markets [21].

The dominance of raw material-based products in traditional Chinese medicinal products exports highlighted the current positioning within the global value chain, where China primarily served as a supplier of unprocessed or minimally processed medicinal materials. This reflected a lower value-added stage in the international trade structure, limiting profit margins and global competitiveness. The significantly higher AAPC observed in plant extracts and TCM health products suggested a growing market demand for semi-processed and functional health products, indicating potential opportunities for industrial upgrading. To enhance value chain positioning, strategic efforts should focus on increasing the export proportion of high-value-added products, such as Chinese patent medicines and standardized TCM health products. This shift could be achieved through investments in technological innovation, compliance with international regulatory standards, and the development of globally recognized traditional Chinese medicinal products brands. Additionally, leveraging digital trade platforms and cross-border e-commerce could facilitate broader market access and promote the internationalization of high-value products.

### 2. Trade associated factors analysis

(1) The growth of China’s GDP was found to have a positive association with the export. This relationship reflected not only the strengthening of domestic economic capacity, but also the advancement and innovation in Chinese medicinal materials cultivation and extraction technologies. Such improvements enhanced the quality and international competitiveness of traditional Chinese medicinal products, aligning with findings from previous studies [15,16].
(2) The GDP of the export country or region’ growth was found to have a positive association with the export. The expansion and economic development of these countries or regions increased their demand for traditional Chinese medicinal products. As global economic integration deepened, the rising demand for natural and health-oriented products across various countries further stimulated the export. This finding was consistent with results from previous studies [15,16].
(3) The capital distance between the export country or region and China was found to have a negative association with the export. Greater distances between capitals resulted in higher transportation costs and extended transit times, which could elevate the overall cost of the export. This increased cost might compromise traditional Chinese medicinal product quality and diminish market competitiveness, thereby constraining the export potential. This finding aligned with the results of previous studies [15,16].
(4) The population of the export country or region was found to have a positive association with the export. A larger population signified a broader market capacity and heightened consumer demand, thereby expanding the market potential for traditional Chinese medicinal products and facilitating their export. This finding was consistent with the results of the previous study [16].
(5) Having a common border with China was found to have a negative association with the export. This finding contradicted results from the previous study [16], which suggested that common borders facilitated trade by providing easier transportation and fostering similar living habits. This discrepancy might arise from the broader sample of countries and regions in this study, which could capture the increased complexity of the contemporary international trade environment. Additionally, the dynamics of global economic integration, the proliferation of regional trade agreements, and the advancements in trade transportation technology might have diminished the traditional benefits of common borders.
(6) Having a common language with China was found to have a positive association with the export, aligning with the findings of the previous study [22]. A common language facilitated smoother trade interactions and reflected a common cultural background, which enhanced the understanding and acceptance of TCM within the export country or region. This cultural affinity contributed to greater appeal and acceptance of traditional Chinese medicinal products, thereby promoting the export.
(7) Being a landlocked country or region was found to have a negative association with the export, consistent with the results of the previous study [16]. Landlocked countries or regions often faced higher trade costs due to challenging terrain and restricted transportation routes, which directly increased the difficulty and expense of the export. In contrast, non-landlocked countries benefited from more accessible maritime transport, which enhanced the efficiency of shipping and thus facilitated the export.
(8) Having trade agreements with China had a negative association with the trade inefficiency term, thereby promoting the export. This finding aligned with the previous study [15]. Trade agreements could minimize trade friction between countries or regions, reducing trade costs and enhancing trade efficiency through mechanisms such as tariff reductions and streamlined customs procedures, thereby supporting increased the export.
(9) Pharmaceutical products tariffs had a positive association with the trade inefficiency term, indicating that higher tariffs impeded the export. This finding was consistent with the previous study [16]. Tariffs impacted market supply and demand by altering the cost structure of export products. Elevated tariffs reduce the price competitiveness of traditional Chinese medicinal products in the international market, leading to higher consumer costs in the export country or region. This, in turn, diminished consumer demand and adversely affected the export.

In addition to tariffs, Non-Tariff Barriers (NTB) are also closely associated with the export efficiency of traditional Chinese medicinal products. These barriers include stringent quality control standards, complex registration and certification procedures, and restrictions on the use of certain medicinal ingredients. Many countries impose rigorous regulatory requirements on traditional Chinese medicinal products, such as Good Manufacturing Practice (GMP) certification, clinical trial data for efficacy verification, and compliance with pharmacopoeial standards, which can significantly increase the time and cost of market entry. Furthermore, cultural and regulatory differences in the acceptance of herbal medicine formulations may further restrict trade, as some active ingredients commonly used in traditional Chinese medicinal face restrictions in certain regions. These NTBs, in conjunction with high tariff rates, can compound trade inefficiencies, making it more difficult for traditional Chinese medicinal products to penetrate international markets. Future research could explore the relative impact of various NTBs and potential strategies for regulatory harmonization to facilitate smoother international trade.

(10) Cultural distance from China had a positive association with the trade inefficiency term, thereby hindering the export. This finding aligned with the previous study [16]. Greater cultural distance entailed more pronounced differences in customs, morals, and values between the two countries or regions. Such cultural discrepancies often lead to challenges in understanding and accepting TCM, thereby affecting their demand for traditional Chinese medicinal products.
(11) The overseas registration status of Chinese patent medicines had a positive association with the trade inefficiency term, thereby hindering the export. This result deviated from expectations. Previous studies had not examined this variable. The relatively small proportion of Chinese patent medicines within the total export volume might have masked or diluted the impact of their overseas registration status on overall export figures. Additionally, the diversity among Chinese patent medicines, with varying registration complexities, market acceptance, and promotional strategies, suggested that the registration status of individual medicines could not be broadly representative, potentially affecting the results.
(12) The presence of TCM institutions in the export country or region had a positive association with the trade inefficiency term, thereby hindering the export. This finding was contrary to expectations and differed from the prior research, which had largely focused on the impact of Confucius Institutes [7]. Previous studies had not explored other overseas institutions, such as TCM centers, TCM hospitals and university branches. The establishment of TCM institutions abroad did not necessarily guarantee an unimpeded flow of traditional Chinese medicinal products. The export process remained constrained by various factors, including quality standards, policy barriers, cultural acceptance, and market demand.

A more plausible explanation for the counterintuitive findings regarding TCM institutions and trade inefficiency is the presence of lag effects in the impact of TCM institutions on export facilitation. The establishment of TCM institutions may take time to generate a tangible increase in demand for traditional Chinese medicinal products, as cultural integration, regulatory alignment, and market expansion efforts require gradual development. Additionally, non-linear relationships could exist, wherein the role of TCM institutions in exports follows a threshold effect-initially facing regulatory and cultural resistance but eventually contributing positively as familiarity and acceptance grow. These constraints likely explained the discrepancy between the study’s findings and initial expectations.

### 3. Policy recommendations

Based on the analysis of the trends and factors associated with the export of traditional Chinese medicinal products, we proposed the following six policy recommendations:

#### (1) Enhance the transportation network for Chinese medicinal product trade.

The analysis indicated that factors such as capital distance, common borders, and landlocked status were associated with the export of traditional Chinese medicinal products, underscoring the importance of trade transportation. A successful example is the China-Europe Railway Express, which has significantly reduced transportation costs and transit time for traditional Chinese medicinal products exports to European markets [23]. Additionally, the China-Laos Railway, launched in 2021, has enhanced trade connectivity between China and Southeast Asia, facilitating faster and more efficient traditional Chinese medicinal products exports [24]. The *Belt and Road* proposal, which integrates international transportation networks into its cooperative framework, provides a strategic framework for expanding the global trade routes for traditional Chinese medicinal products. Initiatives such as the China-Pakistan Railway and the China-Thailand Railway exemplify cross-border transportation projects that can enhance the international export channels for traditional Chinese medicinal products. By addressing geographic barriers, simplifying export processes, facilitating resource allocation and market integration [15], these measures could reduce export transportation difficulties and costs, improve transportation efficiency. To further improve the transportation network for traditional Chinese medicinal products exports, it is essential to establish dedicated multimodal transport hubs in major producing regions, integrating rail, maritime, and air transport to ensure seamless logistics, ultimately promoting the international development of traditional Chinese medicinal products.

#### (2) Strengthen international cooperation in the trade of traditional Chinese medicinal products.

The findings revealed that pharmaceutical products tariffs and trade agreements were associated with the export. Leveraging the advantages of the *Belt and Road* proposal and the Regional Comprehensive Economic Partnership (RCEP) is crucial [5,7]. A case in point is China’s ongoing collaboration with Thailand in the field of traditional medicine, which has promoted mutual understanding and progress toward regulatory alignment for traditional Chinese medicinal products [25]. Similarly, China’s agreements with Hungary and the Czech Republic have promoted the integration of TCM into local healthcare systems [26]. These frameworks present valuable opportunities for advancing the export trade. It is essential to forge and enhance trade relationships with partner countries and regions, actively promoting collaborative programs and projects with the traditional Chinese medicinal industry. Concurrently, support for the domestic traditional Chinese medicinal industry should be bolstered through policy guidance [6], financial backing, and technological innovation. Such measures could facilitate the internationalization of the traditional Chinese medicinal industry and foster the complementarity in traditional medicinal resources on a global scale, achieving mutual benefits and a win-win outcome.

#### (3) Enhance the international dissemination of TCM culture.

The analysis indicated that cultural distance and common language were associated with the export. The differences between TCM and western medicine systems present challenges for the global acceptance of TCM. A notable initiative is the 2024 China-ASEAN TCM Industry Exchange and Promotion Conference, which had advanced the presence of TCM practices in Southeast Asia through academic exchanges, cultural engagement, and medical collaboration [27]. Additionally, the joint celebration of the 5th World TCM Day by the France-China Center for TCM and the China Cultural Center in Paris, had enhanced the visibility and credibility of TCM among European audiences [28]. It is essential to leverage existing international cultural exchange platforms, such as the China-ASEAN Cultural Exchange Year, to advance the global dissemination of TCM culture [4]. Additionally, efforts should be made to utilize internationally recognized languages to promote TCM, including the urgent need to develop of a standardized international terminology system. This approach will facilitate broader acceptance and understanding of TCM concepts. The preventive and holistic healthcare principles inherent in TCM align well with contemporary health paradigms. Thus, promoting TCM through eco-health tourism, including acupuncture, qigong, taijiquan, therapeutic massage, dietary therapies, and hot spring baths, could enhance international acceptance [29].

#### (4) Establish overseas TCM institutions.

Despite the unexpected findings regarding the association with overseas TCM institutions on export, these institutions remain a crucial avenue for disseminating TCM culture. There is a pressing need to establish and enhance overseas TCM institutions, such as TCM centers, Confucius Institutes for TCM, branches of TCM universities, and TCM hospitals. These institutions serve as essential bridges to facilitate academic exchanges, foster international collaborations, and provide platforms for integrating TCM with modern medical practices. A successful example is the Confucius Institute for TCM at London South Bank University, which offers TCM courses, research collaborations, and clinical services [30]. Additionally, China’s partnerships with African countries have led to the establishment of TCM hospitals, such as the China-Uganda Friendship Hospital, where TCM treatments are integrated into local healthcare systems [31]. The improper use of traditional Chinese medicinal products, absent the guidance of TCM theories, may result in limited efficacy or adverse effects. To address these issues, overseas TCM institutions should be leveraged as platforms for integrating TCM with modern medical practices. Focus on advancing medical technology, equipment, and talent, and enhance the demand for traditional Chinese medicinal products. This approach ensures of traditional Chinese medicinal products’ safety and efficacy and promotes their international recognition [29].

#### (5) Optimize the export structure of traditional Chinese medicinal products.

The findings of the association with the overseas registration status of Chinese patent medicines on export were contrary to expectations. This discrepancy might be attributed to the imbalanced export structure of traditional Chinese medicinal products. The export predominantly comprises low-value-added items such as Chinese medicinal materials and decoction pieces, plant extracts. Conversely, high-value-added products, including Chinese patent medicines and TCM health products, represent a minor fraction of the total export value. Optimizing the export structure and enhancing the export of high-value-added products is crucial for improving traditional Chinese medicinal products’ international competitiveness and market share. A noteworthy case is Japan’s success in promoting Kampo medicine, which has been integrated into the national healthcare system and exported as pharmaceutical-grade products [19]. Similarly, South Korea’s export of ginseng-based functional health products has demonstrated how investment in technological innovation and industrial upgrading can enhance competitiveness [32]. Leveraging advanced technologies such as the Internet, big data, and artificial intelligence could significantly enhance the TCM industry’s technological integration and industrial upgrading [3]. Additionally, developing healthcare products derived from medicine and food sources should be prioritized to secure a competitive position in the international market.

#### (6) Promote the standardization of traditional Chinese medicinal products.

Standardization and alignment with international policies are crucial for advancing the international development of traditional Chinese medicinal products. The divergence between TCM and western medicine systems poses a significant challenge for the international acceptance of traditional Chinese medicinal products. To address this issue, it is imperative to advance the standardization of traditional Chinese medicinal products. Increase investment in herb cultivation, harvesting, processing, logistics, traceability, and quality inspection. Among them, establishing production bases that comply with Good Agricultural Practice (GAP) standards and implementing Good Manufacturing Practice (GMP) standards are not just steps, but essential ones to ensure the safety and efficacy of products [3]. A successful model is Germany’s Commission E, which established regulatory standards for herbal medicine, facilitating the international acceptance [33]. Similarly, Japan’s rigorous Kampo medicine registration system has allowed its herbal products to be sold as prescription drugs in many countries [34]. Furthermore, extensive international registration and certification are necessary to align with global market demands. Efforts should be made to register and list traditional Chinese medicinal products through various channels, including traditional botanicals, over-the-counter drugs, and prescription medications. By ensuring compliance with international standards and securing appropriate certifications, traditional Chinese medicinal products could gain entry into the international market, thereby facilitating their global expansion and acceptance.

## Limitation

In the joinpoint regression analysis, the study was limited to a decade of data, which constrained the number of joinpoints identified for subcategories of traditional Chinese medicinal products. This limitation might not fully capture the comprehensive development trends in trade. The stochastic frontier analysis and trade inefficiency analysis revealed three factors with results contrary to expectations, likely due to sample size limitations. It is crucial to overcome these constraints to comprehensively assess the variables’ association, leading to a better understanding of the trade trends.

Additionally, due to the technical constraints of the Frontier 4.1 software used, we were unable to incorporate instrumental variable techniques to address the potential endogeneity of key explanatory variables such as GDP. This limitation may have introduced bias in the estimated coefficients. Moreover, the software does not support panel data structures or methods that account for cross-sectional dependence. As a result, we could not explicitly control for common shocks or unobserved interdependencies among countries.

Future studies should aim to expand the sample size and include data from additional years to address these issues. Extending the period would potentially increase the number of joinpoints detected in regression analysis, thereby enhancing the comprehensiveness and accuracy of trend assessments. A larger sample size would also provide a more robust information base for stochastic frontier analysis, mitigate biases associated with limited data, and facilitate the discovery of more profound and nuanced associated factors. What’s more, future research should adopt more flexible econometric platforms such as Stata or R, which allow for the integration of instrumental variable methods, fixed effects, and cross-sectional dependence correction techniques. Such enhancements would improve the robustness and causal interpretability of the model, thereby providing stronger empirical support for policy recommendations related to the international recognition of TCM.

## Conclusion

This study employed joinpoint regression to analyze the export value trends of traditional Chinese medicinal products from 2013 to 2022. The findings indicated a general upward trend in the export value. However, the export structure remained suboptimal. Specifically, over 80% of exports comprised raw material-based products, such as Chinese medicinal materials and decoction pieces, as well as plant extracts. Their export value increased significantly. Conversely, high-value-added products, including Chinese patent medicines and TCM health products, constituted a relatively minor share of total exports. Among them, the export value of Chinese patent medicines exhibited slow growth. Stochastic frontier analysis and trade inefficiency analysis were conducted for 182 countries and regions from 2020 to 2022 to examine further the factors associated with traditional Chinese medicinal products export. The results revealed that several factors were positively associated with exports, including China’s GDP, the GDP of the export country or region, the population of the export country or region, common language, trade agreements. Conversely, factors such as capital distance, common borders, being a landlocked country or region, tariffs on pharmaceutical products, cultural distance, the overseas registration status of Chinese patent medicines, and overseas TCM institutions were negatively associated with exports. Notably, the effects of common borders, the overseas registration status of Chinese patent medicines, and overseas TCM institutions were contrary to expectations, which might be attributable to sample size limitations. Finally, this study proposed policy recommendations based on these findings to address the current challenges and enhance the international recognition of traditional Chinese medicinal products.

## Supporting information

S1 Table Sources of associated factors and expected results of traditional Chinese medicinal products export. (DOCX)

S2 Table Results of hypothesis testing for stochastic frontier analysis. (DOCX)

S3 Table Robustness tests of trade inefficiency model. (DOCX)

S4 File Data availability statement. (DOCX)

## Abbreviations

AAPC: Annual Average Percent Change
APC: Annual Percent Change
CAGR: Compound Annual Growth Rate
CCCMHPIE: China Chamber of Commerce for Import and Export of Medicines and Health Products
CEPII: Le Centre d’Études Prospectives Et d’Informations Internationales
CIs: Confidence Intervals
DEA: Data Envelopment Analysis
GAP: Good Agricultural Practice
GDP: Gross Domestic Product
GMP: Good Manufacturing Practice
HS: Harmonized System
LR: Likelihood Ratio
MLE: Maximum Likelihood Estimate
NCI: National Cancer Institute
NTB: Non-Tariff Barriers
OLS: Ordinary Least Squares
RCEP: Regional Comprehensive Economic Partnership
RTA: Regional Trade Agreement
SFA: Stochastic Frontier Analysis
TCM: Traditional Chinese Medicine
TFP: Total Factor Productivity Analysis
VIF: Variance Inflation Factor
WTO: World Trade Organization
