# Supplementary material for: Factors associated with the export of traditional Chinese medicinal products: A stochastic frontier analysis
Source: PLoS One. 2025 Jul 9;20(7):e0326422. doi: 10.1371/journal.pone.0326422 (PMC12240354; doi:10.1371/journal.pone.0326422)
Supplement: S1 Table — (DOCX) [file pone.0326422.s001.docx]

**S1 Table. Sources of associated factors and expected results of traditional Chinese medicinal products export.**

| **Model** | **Variable** | **Description** | **Source** | **Expected Result** |
| --- | --- | --- | --- | --- |
| SFA | *gdp* | the GDP of the export country or region | World Bank official website [1] | positive correlation |
|  | *cgdp* | China’s GDP | World Bank official website [1] | positive correlation |
|  | *pop* | the population of the export country or region | World Bank official website [2] | positive correlation |
|  | *cpop* | China’s population | World Bank official website [2] | negative correlation |
|  | *dis* | the capital distance between the export country or region and China | Le Centre d’études prospectives et d’informations internationales (CEPII) official website [3] | negative correlation |
|  | *bor* | the presence of a common border with China | CEPII official website [3] | positive correlation |
|  | *lan* | the presence of a common language with China | CEPII official website [4] | positive correlation |
|  | *loc* | the landlocked status of the export country or region | CEPII official website [3] | negative correlation |
| Trade Inefficiency Analysis | *wto* | the existence of a trade agreement with China | Regional Trade Agreements database [5] | negative correlation |
|  | *cus* | pharmaceutical products tariffs in the export country or region | Tariff analysis online facility provided by World Trade Organization (WTO) [6] | positive correlation |
|  | *cul* | cultural distance from China | Hofstede official website [7] | positive correlation |
|  | *pat* | the overseas registration status of Chinese patent medicines | Web search [8-19] | negative correlation |
|  | *ins* | the presence of TCM institutions in the export country or region | Web search [20-37] | negative correlation |

Sources:

1. World Bank Group. GDP (current US$). <https://data.worldbank.org.cn/indicator/NY.GDP.MKTP.CD>. Accessed on 2 Jun 2024.

2. World Bank Group. [Population, total.](https://data.worldbank.org/indicator/SP.POP.TOTL) <https://data.worldbank.org.cn/indicator/SP.POP.TOTL>[. Accessed on 2 Jun 2024.](https://data.worldbank.org/indicator/SP.POP.TOTL)

3. Recherche Et Expertise Sur L’Economie Mondiale. Geo Dist. <http://www.cepii.fr/CEPII/fr/bdd_modele/bdd_modele_item.asp?id=6>. Accessed on 2 Jun 2024.

4. Recherche Et Expertise Sur L’Economie Mondiale. Language. <http://www.cepii.fr/CEPII/fr/bdd_modele/bdd_modele_item.asp?id=19>. Accessed on 2 Jun 2024.

5. Regional Trade Agreements Database. RTAs in force. <http://rtais.wto.org/UI/PublicMaintainRTAHome.aspx>. Accessed on 2 Jun 2024.

6. World Trade Organization, Organizacion Mundial Del Comercio, Organisation Mondiale Du Commerce. Tariff Analysis Online facility provided by WTO. <http://tao.wto.org/default.aspx>. Accessed on 7 Jun 2024.

7. Hofstede, The Culture Factor Group. Country Comparison Tool. <https://www.hofstede-insights.com/country-comparison-tool?countries>. Accessed on 11 Jun 2024.

8. Guangzhou Baiyunshan Pharmaceutical Holdings Co., Ltd. Two products of Guangzhou Baiyunshan obtained Russian registration certificates, accelerating the pace of internationalization of Chinese medicine. <https://mp.weixin.qq.com/s?__biz=MzA3NTM5MjEzOA==&mid=2649952705&idx=1&sn=df835e16aafb610c2c4e2dabb157d931&chksm=87768424b0010d32f4625769b767a3d4a78f5543d986f47564917308a88760ecfc916bbfdb31&scene=27>. Accessed on 15 Jun 2024.

9. Health Commission of Sichuan Province. Di Ao Cardiovascular Capsules was Registered in the European Union. [https://fanyi.baidu.com/ mtpe-individual/multimodal?query=%E6%AC%A7%E7%9B%9F&lang=zh2en.](https://fanyi.baidu.com/mtpe-individual/multimodal?query=%E6%AC%A7%E7%9B%9F&lang=zh2en.) Accessed on 15 Jun 2024.

10. China News Net. Thirty Years of Yiling Pharmaceutical: Insist on the Bottom Line of Quality and Safety, Promote the Development of Traditional Chinese Medicine through Innovation. [https://www.china news.com.cn/m/cj/2022/06-17/9782277.shtml#backtop.](https://www.chinanews.com.cn/m/cj/2022/06-17/9782277.shtml#backtop.) Accessed on 17 Jun 2024.

11. Guangzhou Xiangxue Pharmaceutical Co., Ltd. Xiangxue Antiviral Oral Solution Received Drug Approval for Upper Respiratory Tract Infections in Canada. [https://mp.weixin.qq.com/s?__biz=MzA5OTA 1OTAzNA==&mid=402898572&idx=1&sn=1b6754823bb0da119a3747f3dbbd16bc&chksm=0d7f0ab93a0883af52b5ec285f44dbc16c6ec70353f755548851c47fa9ed57372d9e72386b42&scene=27.](https://mp.weixin.qq.com/s?__biz=MzA5OTA1OTAzNA==&mid=402898572&idx=1&sn=1b6754823bb0da119a3747f3dbbd16bc&chksm=0d7f0ab93a0883af52b5ec285f44dbc16c6ec70353f755548851c47fa9ed57372d9e72386b42&scene=27.) Accessed on 15 Jun 2024.

12. Xueqiu. Tianshili: Announcement on the Approval of Registration of Danshen Capsule as Botanical Drug in the Netherlands. <https://xueqiu.com/3491303582/63796692.> Accessed on 17 Jun 2024.

13. China News Net. Chinese Medicine Dan Ning Pian Tablets Successfully Exported to Canada, Entering the International Mainstream Drug Market. [https://baijiahao.baidu.com/s?id=1746759997440130022&wfr=spider &for=pc.](https://baijiahao.baidu.com/s?id=1746759997440130022&wfr=spider&for=pc.) Accessed on 15 Jun 2024.

14. China Economic Net. China’s Banlangen Products was Approved for the First Time in the United Kingdom. [http://www.ce.cn/cysc/sp/info/ 201708/17/t20170817_25085483.shtml](http://www.ce.cn/cysc/sp/info/201708/17/t20170817_25085483.shtml). Accessed on 15 Jun 2024.

15. West China Hospital of Sichuan University. Prof. Li Tingqian’s Lemai Granules was Approved for Marketing in Canada. [https://www.wchscu. cn/comprehensive/36536.html.](https://www.wchscu.cn/comprehensive/36536.html.) Accessed on 17 Jun 2024.

16. China Traditional Chinese Medicine Newspaper Official Account. What Should I Do if I Have A Headache or Stiff Neck? Yu Feng Ning Xin Tablet: Promoting Blood Circulation and Relieving Pain. <https://mp.weixin.qq.com/s?__biz=MzUyMjUzMjU2Mg==&mid=2247571718&idx=3&sn=25916d1f2f11d795518a9f519d3681b6&chksm=f9c9fabbcebe73ad33b83b47a43f688d68501426b2f28b57baf40f1be0f71ecf7870284173fc&scene=27.> Accessed on 17 Jun 2024.

17. Beijing News. Tianshili Xiao Yao Tablets was Approved in the Netherlands. [https://baijiahao.baidu.com /s?id=1710419591595737585& wfr=spider&for=pc.](https://baijiahao.baidu.com/s?id=1710419591595737585&wfr=spider&for=pc.) Accessed on 18 Jun 2024.

18. China News Net. Yiling Pharmaceuticals: Lianhua Clear Cough Tablets Received Registration Approval for Chinese Patent Medicine in Singapore. [https://baijiahao.baidu.com/s?id=1752014589698936136&wfr =spider&for=pc.](https://baijiahao.baidu.com/s?id=1752014589698936136&wfr=spider&for=pc.) Accessed on 18 Jun 2024.

19. China Daily Net. Hua Shi Bai Du Granules was Recognized by the United Arab Emirates, Chinese Medicine Helped to Treat COVID-19. <https://baijiahao.baidu.com/s?id=1680955411251181234&wfr=spider&for=pc.> Accessed on 18 Jun 2024.

20. Beijing University of Chinese Medicine. General Introduction. [https://www.bucm.edu.cn/xxgk1/xxjj1/ index.htm.](https://www.bucm.edu.cn/xxgk1/xxjj1/index.htm.) Accessed on 20 Jun 2024.

21. Shanghai University of Traditional Chinese Medicine. School Introduction. <https://www.shutcm.edu.cn/6550/> list.htm. Accessed on 20 Jun 2024.

22. China Academy of Chinese Medical Sciences. PROFILE. <https://www.cacms.ac.cn/his_address/detail/> 1008.html. Accessed on 20 Jun 2024.

23. Nanjing University of Chinese Medicine. Introduction of NJUCM. <https://www.njucm.edu.cn/6731/list.htm.> Accessed on 20 Jun 2024.

24. Zhejiang Chinese Medical University. Our Profile. [https://www.zcmu. edu.cn/xqzl/xxgk1.htm.](https://www.zcmu.edu.cn/xqzl/xxgk1.htm.) Accessed on 20 Jun 2024.

25. North China University of Science and Technology. School Introduction. [https://www.ncst.edu.cn/col/ 1511411853275/index.html.](https://www.ncst.edu.cn/col/1511411853275/index.html.) Accessed on 20 Jun 2024.

26. Xinhua News Agency. Confucius Institute for Traditional Chinese Medicine Established in Romania. <https://baijiahao.baidu.com/s?id=1797391013326528264&wfr=spider&for=pc.> Accessed on 20 Jun 2024.

27. Hebei University of Chinese Medicine. School Profile. [https://www.hebcm.edu.cn/col/1463637802676/ index.html.](https://www.hebcm.edu.cn/col/1463637802676/index.html.) Accessed on 20 Jun 2024.

28. Liaoning University of Traditional Chinese Medicine. School Introduction. [https://www.lnutcm.edu.cn/xxgk/ xxjj.htm.](https://www.lnutcm.edu.cn/xxgk/xxjj.htm.) Accessed on 20 Jun 2024.

29. Chengdu University of Traditional Chinese Medicine. Introduction. <https://www.cdutcm.edu.cn/xxgk/xxjj.> Accessed on 20 Jun 2024.

30. Fujian University of Traditional Chinese Medicine. School Introduction. [https://www.fjtcm.edu.cn/1294/ list.htm.](https://www.fjtcm.edu.cn/1294/list.htm.) Accessed on 20 Jun 2024.

31. Shandong University of Traditional Chinese Medicine. School Introduction. [https://www.sdutcm.edu.cn/xxgk1/ xxjj.htm.](https://www.sdutcm.edu.cn/xxgk1/xxjj.htm.) Accessed on 21 Jun 2024.

32. Hunan University of Chinese Medicine. School Introduction. <https://www.hnucm.edu.cn/xxgk/xxjj.htm.> Accessed on 21 Jun 2024.

33. Anhui University of Chinese Medicine. School Introduction. <https://www.ahtcm.edu.cn/xxgk/xxjj.htm.> Accessed on 21 Jun 2024.

34. Jiangxi University of Chinese Medicine. School Introduction. <https://www.jxutcm.edu.cn/info/1050/16932.htm.> Accessed on 21 Jun 2024.

35. Shaanxi University of Chinese Medicine. School Introduction. <http://www.sntcm.edu.cn/xxgk/xygk/13539.htm.> Accessed on 21 Jun 2024.

36. Yunnan University of Chinese Medicine. School Introduction. [https://www.ynucm.edu.cn/xxgk/yzjj/index. shtml.](https://www.ynucm.edu.cn/xxgk/yzjj/index.shtml.) Accessed on 21 Jun 2024.

37. Tianjin University of Traditional Chinese Medicine. School Introduction. [https://news13.tjutcm.edu.cn/info/ 1636/7910.htm.](https://news13.tjutcm.edu.cn/info/1636/7910.htm.) Accessed on 21 Jun 2024.
