# Supplementary material for: Factors associated with the export of traditional Chinese medicinal products: A stochastic frontier analysis
Source: PLoS One. 2025 Jul 9;20(7):e0326422. doi: 10.1371/journal.pone.0326422 (PMC12240354; doi:10.1371/journal.pone.0326422)
Supplement: S2 Table — (DOCX) [file pone.0326422.s002.docx]

**S2 Table. Results of hypothesis testing for stochastic frontier analysis.**

| **Original Hypothesis** | **Constrained Model（H0）** | **Unconstrained Model（H1）** | **LR Statistical Values** | **Threshold Value of 1%** | **Test Conclusion** | **Degrees of Freedom** | **Conclusion** |
| --- | --- | --- | --- | --- | --- | --- | --- |
| Trade inefficiency term was absent | -20.6872 | 369.7711 | 780.9166 | 10.501 | Reject the original hypothesis | 3 | Trade inefficiency term was exist |
| Trade inefficiency term was static over time | -20.6872 | 415.7678 | 872.9100 | 8.273 | Reject the original hypothesis | 2 | Used time-varying model |
| Excluded the *gdp* variable | -56.2000 | 414.7665 | 941.9330 | 10.501 | Reject the original hypothesis | 3 | Kept the variable |
| Excluded the *cgdp* variable | -20.730255 | 433.4966 | 908.4537 | 10.501 | Reject the original hypothesis | 3 | Kept the variable |
| Excluded the *dis* variable | -32.8474 | 329.4406 | 724.5760 | 10.501 | Reject the original hypothesis | 3 | Kept the variable |
| Excluded the *pop* variable | -21.0853 | 322.3389 | 686.8484 | 10.501 | Reject the original hypothesis | 3 | Kept the variable |
| Excluded the *bor* variable | -21.3138 | 423.9087 | 890.4450 | 10.501 | Reject the original hypothesis | 3 | Kept the variable |
| Excluded the *lan* variable | -47.4768 | 402.8993 | 900.7522 | 10.501 | Reject the original hypothesis | 3 | Kept the variable |
| Excluded the *loc* variable | -29.0747 | 433.3624 | 924.8742 | 10.501 | Reject the original hypothesis | 3 | Kept the variable |
