# Supplementary material for: Factors associated with the export of traditional Chinese medicinal products: A stochastic frontier analysis
Source: PLoS One. 2025 Jul 9;20(7):e0326422. doi: 10.1371/journal.pone.0326422 (PMC12240354; doi:10.1371/journal.pone.0326422)
Supplement: S3 Table — (DOCX) [file pone.0326422.s003.docx]

**S3 Table. Robustness tests of trade inefficiency model.**

| **Variable** | | **Model 1** | | | **Model 2** | | | **Model 3** | | | **Model 4** | | | **Model 5** | | |
| --- | --- | --- | --- | --- | --- | --- | --- | --- | --- | --- | --- | --- | --- | --- | --- | --- |
|  |  | **Coefficient** | **Standard-Error** | **t-Ratio** | **Coefficient** | **Standard-Error** | **t-Ratio** | **Coefficient** | **Standard-Error** | **t-Ratio** | **Coefficient** | **Standard**  **-Error** | **t-Ratio** | **Coefficient** | **Standard**  **-Error** | **t-Ratio** |
| SFA | constant | 0.1253 | 0.9613 | 0.1304 | 0.0760 | 1.0090 | 0.0753 | -0.1922 | 0.3259 | -0.5897 | -0.0604 | 0.0941 | -0.6416 | 0.2136*** | 0.0183 | 11.6509 |
|  | *lngdp* | -0.0005 | 0.0010 | -0.5037 | 0.0002 | 0.0038 | 0.0410 | 0.0016*** | 0.0004 | 4.6661 | 0.0005*** | 0.0001 | 5.3739 | 0.0001 | 0.0001 | 0.3992 |
|  | *lncgdp* | -0.0014 | 0.0326 | -0.0431 | -0.0047 | 0.0334 | -0.1407 | 0.0050 | 0.0106 | 0.4681 | 0.0015 | 0.0032 | 0.4787 | -0.0076*** | 0.0007 | -11.2694 |
|  | *lndis* | -0.0095 | 0.0061 | -1.5634 | 0.0021 | 0.0082 | 0.2489 | -0.0056*** | 0.0019 | -2.9966 | -0.0026*** | 0.0008 | -3.3937 | -0.0014*** | 0.0004 | -3.5150 |
|  | *lnpop* | 0.0004 | 0.0017 | 0.2547 | 0.0026 | 0.0042 | 0.6138 | 0.0032*** | 0.0006 | 5.4223 | 0.0014*** | 0.0003 | 4.6617 | 0.0016*** | 0.0002 | 9.5793 |
|  | *lnbor* | -0.0211 | 0.0194 | -1.0826 | -0.0137 | 0.0233 | -0.5881 | -0.0404*** | 0.0083 | -4.8879 | -0.0055*** | 0.0015 | -3.7184 | 0.0035*** | 0.0007 | 4.7977 |
|  | *lnlan* | 0.8707*** | 0.0095 | 91.4223 | 0.7892*** | 0.0235 | 33.6318 | 0.8539*** | 0.0052 | 165.7168 | 0.8601*** | 0.0053 | 160.9178 | 0.8187*** | 0.0069 | 118.7665 |
|  | *lnloc* | -0.0215*** | 0.0054 | -3.9582 | -0.0161 | 0.0220 | -0.7292 | -0.0056** | 0.0023 | -2.4104 | 0.0010 | 0.0011 | 0.9650 | -0.0027*** | 0.0004 | -7.5932 |
| Trade Inefficiency Analysis | constant | -1.8407*** | 0.1037 | -17.7582 | -0.9139*** | 0.0871 | -10.4974 | -4.7523*** | 0.0723 | -65.7663 | -5.8790*** | 0.4318 | -13.6156 | -6.2634*** | 0.6003 | -10.4332 |
|  | *lnwto* | - | - | - | -1.6377 | 0.9990 | -1.6393 | -5.1712*** | 0.1719 | -30.0794 | -6.3554*** | 0.2208 | -28.7837 | -1.0357** | 0.4221 | -2.4537 |
|  | *lncus* | 0.1240*** | 0.0349 | 3.5510 | - | - | - | 0.3649*** | 0.0342 | 10.6586 | 0.2448*** | 0.0382 | 6.4099 | 0.7223*** | 0.0835 | 8.6493 |
|  | *lncul* | 0.6276*** | 0.0723 | 8.6801 | 0.2446*** | 0.0758 | 3.2267 | - | - | - | -0.0318 | 0.0849 | -0.3747 | 1.6477*** | 0.1270 | 12.9709 |
|  | *lnpat* | 1.1938*** | 0.1370 | 8.7148 | 1.0198*** | 0.1447 | 7.0458 | 2.3279*** | 0.1370 | 16.9877 | - | - | - | 5.4729*** | 0.5264 | 10.3963 |
|  | *lnins* | 0.9257*** | 0.0824 | 11.2375 | 0.9170*** | 0.1073 | 8.5463 | 5.5838*** | 0.1116 | 50.0441 | 6.7105*** | 0.4751 | 14.1233 | - | - | - |
|  | σ2 | 0.1565*** | 0.0073 | 21.5530 | 0.0940*** | 0.0073 | 12.8914 | 0.3341*** | 0.0089 | 37.4035 | 0.5282*** | 0.0342 | 15.4306 | 0.5436*** | 0.0518 | 10.4874 |
|  | γ | 0.9999*** | 0.00006 | 17649 | 0.9999*** | 0.0005 | 2216 | 0.9999*** | 0.000007 | 152920 | 0.9999*** | 0.00000003 | 36207433 | 0.9999*** | 0.00000001 | 68572160 |
|  | log likelihood function | 485.0136 | | | 438.1075 | | | 619.8051 | | | 648.4827 | | | 569.1874 | | |
|  | LR test of the one-sided error | 1011.4017 | | | 917.5894 | | | 1280.9846 | | | 1338.3399 | | | 1179.7492 | | |

***, **, * respectively represents 1%, 5% and 10% significance levels.
