# Supplementary material for: Factors associated with the export of traditional Chinese medicinal products: A stochastic frontier analysis
Source: PLoS One. 2025 Jul 9;20(7):e0326422. doi: 10.1371/journal.pone.0326422 (PMC12240354; doi:10.1371/journal.pone.0326422)
Supplement: S4 File — (DOCX) [file pone.0326422.s004.docx]

**S4 File. Data availability statement**

All relevant data supporting the findings of this study are available in the public repositories. Details are provided in the list below.

1. World Bank official website: <https://data.worldbank.org.cn/indicator/NY.GDP.MKTP.CD>

2. World Bank official website: <https://data.worldbank.org.cn/indicator/SP.POP.TOTL>

3. Le Centre d’études prospectives et d’informations internationales (CEPII) official website:

<http://www.cepii.fr/CEPII/fr/bdd_modele/bdd_modele_item.asp?id=6>

4. CEPII official website: <http://www.cepii.fr/CEPII/fr/bdd_modele/bdd_modele_item.asp?id=19>

5. Regional Trade Agreements database: <http://rtais.wto.org/UI/PublicMaintainRTAHome.aspx>

6. Tariff analysis online facility provided by World Trade Organization (WTO): <http://tao.wto.org/default.aspx>

7. Hofstede official website: <https://www.hofstede-insights.com/country-comparison-tool?countries>

8. Web search:

<https://mp.weixin.qq.com/s?__biz=MzA3NTM5MjEzOA==&mid=2649952705&idx=1&sn=df835e16aafb610c2c4e2dabb157d931&chksm=87768424b0010d32f4625769b767a3d4a78f5543d986f47564917308a88760ecfc916bbfdb31&scene=27>

9. Web search:

[https://fanyi.baidu.com/mtpe-individual/multimodal?query=%E6%AC%A7%E7%9B%9F&lang=zh2en](https://fanyi.baidu.com/mtpe-individual/multimodal?query=%E6%AC%A7%E7%9B%9F&lang=zh2en.)

10. Web search:

https://www.china news.com.cn/m/cj/2022/06-17/9782277.shtml#backtop

11. Web search:

[https://mp.weixin.qq.com/s?__biz=MzA5OTA 1OTAzNA==&mid=402898572&idx=1&sn=1b6754823bb0da119a3747f3dbbd16bc&chksm=0d7f0ab93a0883af52b5ec285f44dbc16c6ec70353f755548851c47fa9ed57372d9e72386b42&scene=27](https://mp.weixin.qq.com/s?__biz=MzA5OTA1OTAzNA==&mid=402898572&idx=1&sn=1b6754823bb0da119a3747f3dbbd16bc&chksm=0d7f0ab93a0883af52b5ec285f44dbc16c6ec70353f755548851c47fa9ed57372d9e72386b42&scene=27.)

12. Web search: [https://xueqiu.com/3491303582/63796692](https://xueqiu.com/3491303582/63796692.)

13. Web search: [https://baijiahao.baidu.com/s?id=1746759997440130022 &wfr=spider&for=pc](https://baijiahao.baidu.com/s?id=1746759997440130022&wfr=spider&for=pc.)

14. Web search: [http://www.ce.cn/cysc/sp/info/ 201708/17/t20170817_25085483.shtml](http://www.ce.cn/cysc/sp/info/201708/17/t20170817_25085483.shtml)

15. Web search: [https://www.wchscu. cn/comprehensive/36536.html](https://www.wchscu.cn/comprehensive/36536.html.)

16. Web search:

[https://mp.weixin.qq.com/s?__biz=MzUyMjUzMjU2Mg==&mid=2247571718&idx=3&sn=25916d1f2f11d795518a9f519d3681b6&chksm=f9c9fabbcebe73ad33b83b47a43f688d68501426b2f28b57baf40f1be0f71ecf7870284173fc&scene=27](https://mp.weixin.qq.com/s?__biz=MzUyMjUzMjU2Mg==&mid=2247571718&idx=3&sn=25916d1f2f11d795518a9f519d3681b6&chksm=f9c9fabbcebe73ad33b83b47a43f688d68501426b2f28b57baf40f1be0f71ecf7870284173fc&scene=27.)

17. Web search: [https://baijiahao.baidu.com/s?id=1710419591595737585& wfr=spider&for=pc](https://baijiahao.baidu.com/s?id=1710419591595737585&wfr=spider&for=pc.)

18. Web search: [https://baijiahao.baidu.com/s?id=1752014589698936136&wfr=spider&for=pc](https://baijiahao.baidu.com/s?id=1752014589698936136&wfr=spider&for=pc.)

19. Web search: [https://baijiahao.baidu.com/s?id=1680955411251181234&wfr=spider&for=pc](https://baijiahao.baidu.com/s?id=1680955411251181234&wfr=spider&for=pc.)

20. Web search: [https://www.bucm.edu.cn/xxgk1/xxjj1/index.htm](https://www.bucm.edu.cn/xxgk1/xxjj1/index.htm.)

21. Web search: https://www.shutcm.edu.cn/6550/list.htm

22. Web search: https://www.cacms.ac.cn/his_address/detail/1008.html

23. Web search: [https://www.njucm.edu.cn/6731/list.htm](https://www.njucm.edu.cn/6731/list.htm.)

24. Web search: [https://www.zcmu. edu.cn/xqzl/xxgk1.htm](https://www.zcmu.edu.cn/xqzl/xxgk1.htm.)

25. Web search: [https://www.ncst.edu.cn/col/1511411853275/index.html](https://www.ncst.edu.cn/col/1511411853275/index.html.)

26. Web search: [https://baijiahao.baidu.com/s?id =1797391013326528264&wfr=spider&for=pc](https://baijiahao.baidu.com/s?id=1797391013326528264&wfr=spider&for=pc.)

27. Web search: [https://www.hebcm.edu.cn/col/1463637802676/index.html](https://www.hebcm.edu.cn/col/1463637802676/index.html.)

28. Web search: [https://www.lnutcm.edu.cn/xxgk/xxjj.htm](https://www.lnutcm.edu.cn/xxgk/xxjj.htm.)

29.Web search: [https://www.cdutcm.edu.cn/xxgk/xxjj](https://www.cdutcm.edu.cn/xxgk/xxjj.)

30. Web search: [https://www.fjtcm.edu.cn/1294/list.htm](https://www.fjtcm.edu.cn/1294/list.htm.)

31. Web search: [https://www.sdutcm.edu.cn/xxgk1/xxjj.htm](https://www.sdutcm.edu.cn/xxgk1/xxjj.htm.)

32. Web search: [https://www.hnucm.edu.cn/xxgk/xxjj.htm](https://www.hnucm.edu.cn/xxgk/xxjj.htm.)

33. Web search: [https://www.ahtcm.edu.cn/xxgk/xxjj.htm](https://www.ahtcm.edu.cn/xxgk/xxjj.htm.)

34. Web search:[https://www.jxutcm.edu.cn/info/1050/16932.htm](https://www.jxutcm.edu.cn/info/1050/16932.htm.)

35. Web search: [http://www.sntcm.edu.cn/xxgk/xygk/13539.htm](http://www.sntcm.edu.cn/xxgk/xygk/13539.htm.)

36. Web search: [https://www.ynucm.edu.cn/xxgk/yzjj/index.shtml](https://www.ynucm.edu.cn/xxgk/yzjj/index.shtml.)

37. Web search: [https://news13.tjutcm.edu.cn/info/1636/7910.htm](https://news13.tjutcm.edu.cn/info/1636/7910.htm.)

38. General Administration of Customs of the People's Republic of China: http://stats.customs.gov.cn/
